# Quality of life and caregiver burden among caregivers of children with autism spectrum disorder and neurotypical children: a cross-sectional study

**DOI:** 10.31744/einstein_journal/2026AO2383

**Published:** 2026-08-14

**Authors:** João Victor Soares Rodrigues, Wilton Mitsunari Takeshita, Rafael Arakaki Garcia, Alessandra Marcondes Aranega, Rafael Scaf de Molon, Letícia Helena Theodoro

**Affiliations:** 1 Universidade Estadual Paulista "Júlio de Mesquita Filho" Faculdade de Odontologia Department of Diagnosis and Surgery Araçatuba SP Brazil Department of Diagnosis and Surgery, Faculdade de Odontologia, Universidade Estadual Paulista "Júlio de Mesquita Filho", Araçatuba, SP, Brazil.; 2 Universidade Estadual Paulista "Júlio de Mesquita Filho" Center for Dental Assistance to Persons with Disabilities Faculdade de Odontologia Araçatuba SP Brazil Center for Dental Assistance to Persons with Disabilities (CAOE), Faculdade de Odontologia, Universidade Estadual Paulista "Júlio de Mesquita Filho", Araçatuba, SP, Brazil.; 3 Etec Araçatuba Araçatuba SP Brazil Etec Araçatuba, Araçatuba, SP, Brazil.

**Keywords:** Autism spectrum disorder, Caregivers, Quality of life, Caregiver burden, Dental care, Dental care for persons with disabilities

## Abstract

**Objectives:**

This study aimed to compare quality of life and caregiver burden between caregivers and guardians of children with autism spectrum disorder and those of neurotypical children.

**Methods:**

A total of 124 caregivers participated and were assigned to two groups: autism spectrum disorder (ASD; n=62) and neurotypical (NT; n=62). Participants were recruited from a specialized multiprofessional dental care institution. Data were collected using a sociodemographic questionnaire, the WHOQOL-BREF, and the Zarit Burden Interview. Data were tabulated and analyzed using a 5% significance level.

**Results:**

The mean age of the children was 9.45 years in the ASD Group and 8.29 years in the NT Group. The proportion of boys was higher in the ASD Group than in the NT Group (p<0.001). Regarding functional independence, 69.4% of children with ASD depended on caregivers for activities of daily living, whereas 67.7% of neurotypical children were independent (p<0.001). For oral hygiene, 64.4% of children with ASD required caregiver assistance, whereas 82.3% of neurotypical children performed oral hygiene independently (p<0.001). Overall quality of life did not differ significantly between groups. However, specific items in the physical and psychological domains differed significantly between groups (Q3: p=0.021; Q4: p=0.001; Q26: p=0.003). By contrast, caregiver burden was significantly higher among caregivers of children with autism spectrum disorder than among caregivers of NT children, as reflected by higher total and domain scores (p<0.001).

**Conclusion:**

Although caregivers in both groups reported moderate quality of life, those caring for children with autism spectrum disorder experienced substantially greater caregiver burden. These findings support the need for targeted strategies to reduce caregiver stress and improve well-being in families of children with autism spectrum disorder.

**Brazilian Registry of Clinical Trials**: RBR-4cvz58n and U1111-1299-9942.

## Highlights

■Caregivers of children with autism spectrum disorder reported higher caregiver burden.

■Children with autismo spectrum disorder showed greater dependence in activities of daily living.

■Overall quality of life was similar between groups, but specific items differed.

## In Brief

This cross-sectional study compared quality of life and caregiver burden between caregivers of children with autism spectrum disorder and those of neurotypical children. Although overall quality of life was similar between groups, caregivers of children with autism spectrum disorder reported significantly higher caregiver burden. These findings support the need for targeted strategies to reduce caregiver burden and improve family well-being.

## Graphical abstract

**Figure:**
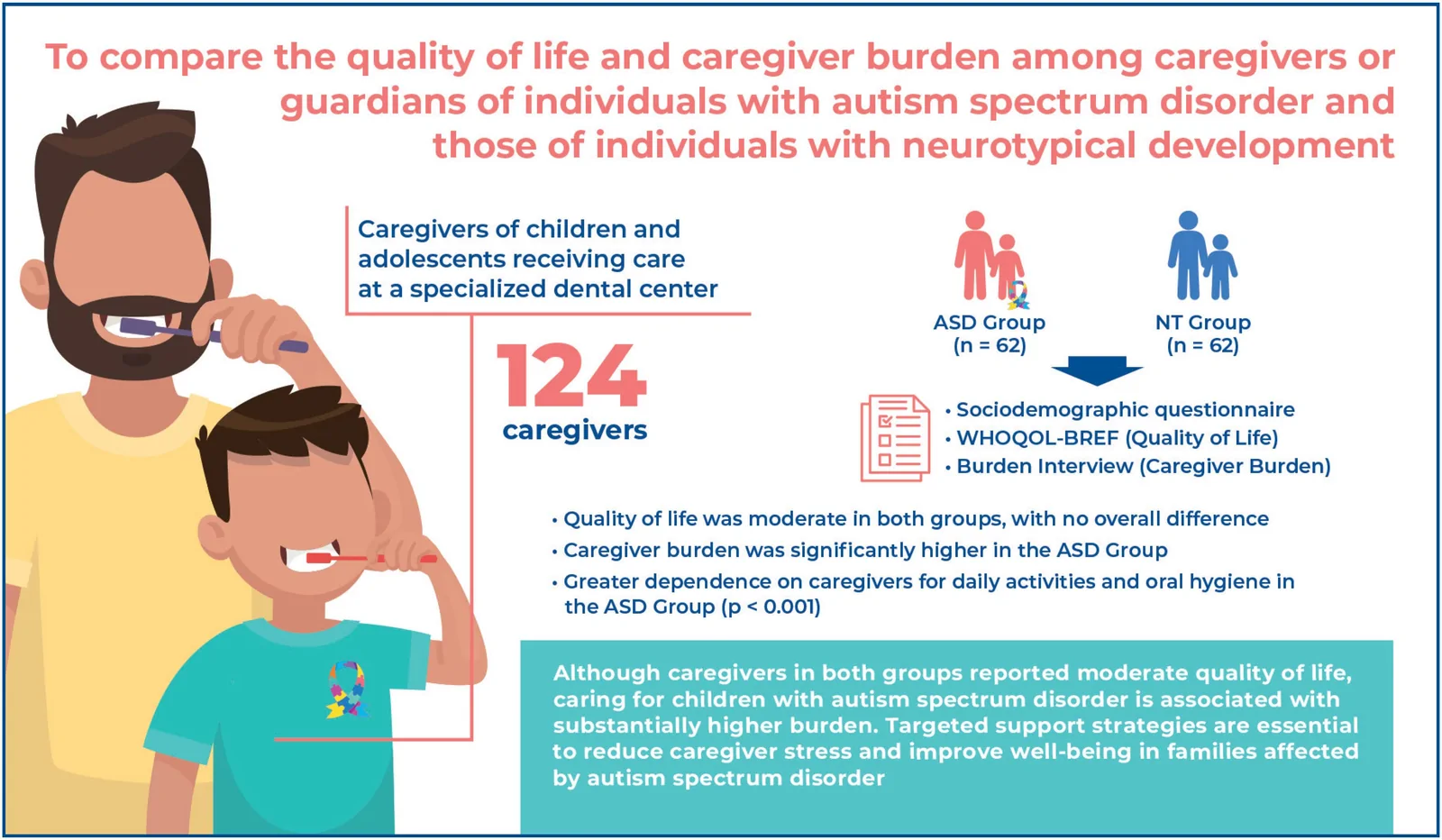

## INTRODUCTION

Autism spectrum disorder (ASD) is a complex neurodevelopmental condition characterized by persistent deficits in social communication and interaction and restricted or repetitive patterns of behavior, interests, or activities.^(1,2)^ These behavioral and cognitive characteristics often emerge in early childhood and persist throughout life, with considerable variability in functional outcomes and adaptive behavior. Clinical presentations vary widely, from individuals who require substantial daily support to those who function independently but continue to experience challenges in social communication and flexibility.

The etiology of ASD is multifactorial, involving genetic, epigenetic, and environmental factors.^(1,3)^ Genetic susceptibility plays a major role, and several chromosomal abnormalities and gene mutations have been identified as contributing factors. Environmental influences, including intrauterine viral infections,^(4)^ toxin exposure, and perinatal complications, may act as triggers or amplifiers in genetically predisposed individuals. Mitochondrial dysfunction has also been implicated and may reduce cellular energy production and impair neurotransmitter release in key brain regions, including the cerebellum, prefrontal cortex, and temporal lobes.^(5,6)^ Together, these mechanisms may contribute to the structural and functional brain alterations associated with the behavioral and cognitive manifestations of ASD.

The reported global prevalence of ASD has increased substantially over the past two decades, partly reflecting greater awareness and improved diagnostic sensitivity.^(7)^ In Brazil, the 2022 Census conducted by the Brazilian Institute of Geography and Statistics (IBGE - *Instituto Brasileiro de Geografia e Estatística*) identified approximately 2.4 million individuals with ASD, representing 1.2% of the population aged two years or older. Among school-aged children six years or older, 760,800 were identified as having ASD, corresponding to 1.7% of the student population and indicating a higher diagnostic prevalence among children and adolescents than among adults.^(8)^ Consistent with global data, ASD is more prevalent in boys, with an estimated male-to-female ratio of approximately 4:1.^(9)^

Caring for an individual with ASD often requires substantial commitment and adaptability. Caregivers, frequently parents or close family members, assist with daily living activities, support communication, manage behavioral challenges, and promote social inclusion throughout the individual's development into adulthood.^(10,11)^ Despite its rewarding aspects, caregiving may involve continuous physical, emotional, and educational demands, which may be compounded by limited access to specialized guidance and social support and may increase stress, anxiety, and depression among caregivers.^(10,12)^ This chronic strain can affect psychological well-being and overall quality of life (QoL).

Caregiver burden encompasses the physical, emotional, social, and financial strain experienced by individuals who provide prolonged care for persons with special needs.^(13,14)^ This burden arises not only from the intensity of caregiving tasks but also from social isolation, role conflicts, and reduced personal time. Over time, these challenges can lead to fatigue, sleep disturbances, somatic symptoms, and poorer physical and mental health, thereby reducing caregivers’ QoL. Chronic caregiving-related stress may also lead caregivers to neglect their own health, including oral health practices, potentially reinforcing the cycle of caregiver burden and poor well-being.^(15,16)^

Studies have reported higher burden among caregivers of individuals with ASD than among caregivers of neurotypical (NT) children.^(11,17)^ This increased burden may reflect the complexity of managing behavioral difficulties, navigating healthcare systems, and advocating for specialized educational and therapeutic services. Caregivers often prioritize the child's well-being, particularly in essential domains such as health and education, even at the expense of their own emotional and physical needs.^(11)^ Although such prioritization may support child health outcomes, it can contribute to cumulative caregiver stress and exhaustion.

Caregiver burden may be further amplified by reduced time for personal, social, and professional activities.^(18)^ Ongoing caregiving demands require emotional resilience, physical endurance, and social support. Without adequate psychoeducational resources and accessible primary healthcare, caregivers may face an increased risk of mental health problems and reduced capacity to provide sustained care. Targeted interventions that offer psychosocial support, stress management, and community-based education may improve caregivers’ well-being and the care provided to individuals with ASD. Designing effective support strategies requires a context-specific understanding of caregivers’ needs, sociodemographic characteristics, and challenges, including how these differ from those of caregivers of NT children.

Although caregiver burden in ASD has been widely documented,^(19-26)^ most studies have been conducted in general medical, psychological, or community-based settings, with limited attention to the specific challenges encountered in dental care environments. Oral health management in children with ASD often requires substantial caregiver involvement because of behavioral difficulties, sensory sensitivities, and reduced autonomy in daily oral hygiene practices. These factors may introduce context-specific stressors that are not fully captured in the broader caregiver burden literature. Moreover, few studies have directly compared caregivers of children with ASD and caregivers of NT children within the same clinical care setting, limiting the ability to distinguish ASD-related caregiving demands from differences in healthcare access. Therefore, evaluating caregiver burden and QoL within a specialized dental care context may clarify how oral health-related demands are associated with caregiver strain and overall well-being.

## OBJECTIVE

The present study aimed to assess and compare quality of life and caregiver burden between caregivers or guardians of children with autism spectrum disorder receiving care at a specialized multiprofessional dental institution and caregivers or guardians of neurotypical children, with emphasis on oral health-related care demands.

## METHODS

### Study design and ethical considerations

This observational, cross-sectional clinical study was conducted at the *Centro de Assistência Odontológica a Pessoas com Deficiência* (CAOE) and the Pediatric Dentistry Clinic of the *Faculdade de Odontologia de Araçatuba*, *Universidade Estadual Paulista* (UNESP), between September 2022 and April 2025. The study was approved by the Human Research Ethics Committee of the School of Dentistry of Araçatuba (CAAE: 59159422.0.0000.5420; # 5.672.313) and conducted in accordance with the Declaration of Helsinki (1975, revised in 2013). The study was also registered in the Brazilian Clinical Trials Registry and the International Clinical Trials Registry Platform. The research protocol followed the Strengthening the Reporting of Observational Studies in Epidemiology (STROBE) guidelines.

### Sample size calculation

The sample size was calculated based on a previous study that assessed QoL among caregivers of individuals with ASD using the WHOQOL-BREF instrument.^(27)^ A minimum sample of 100 caregivers was required to detect a 10-point difference in WHOQOL-BREF scores between groups, with 90% statistical power and a significance level of 0.05. After accounting for a possible 20% attrition rate, 124 participants were included. This calculation was based on QoL outcomes; no a priori power analysis was performed specifically for caregiver burden or multivariable regression analyses.

### Participant selection

Participants were recruited through consecutive sampling, whereby all eligible caregivers attending the participating clinical centers during the study period were invited to participate. A total of 124 caregivers or legal guardians of children were allocated to two groups: the ASD Group (n=62), comprising caregivers of children diagnosed with ASD levels 1, 2, or 3, and the NT Group (n=62), comprising caregivers of children with typical neurodevelopment. All participants received detailed verbal and written information about the study objectives and procedures and provided written informed consent before participation.

The inclusion criteria for the ASD Group were caregivers or guardians of children aged 5-12 years, of either sex, with an established clinical diagnosis of ASD levels 1-3. For the NT Group, inclusion required the absence of any diagnosed neurodevelopmental disorder. The exclusion criteria were caregivers of children with ASD and NT who had aggravating medical comorbidities requiring priority treatment, caregivers with cognitive impairments that prevented questionnaire completion, and caregivers with previously diagnosed psychiatric disorders, to minimize potential confounding in self-reported QoL and burden measures.^(11,26)^

### Data collection

#### Sociodemographic information

A structured questionnaire was administered to collect sociodemographic data, including caregiver age, sex, educational level, marital status, and income. The child's age, sex, and degree of dependence in activities of daily living and oral hygiene were also recorded.^(11,26)^

#### Quality-of-life assessment (WHOQOL-BREF)

Caregivers’ perceived QoL was assessed using the World Health Organization Quality of Life abbreviated questionnaire (WHOQOL-BREF), which was derived from the original WHOQOL-100 instrument. This validated instrument has been culturally adapted for use in Brazil.^(28)^ The WHOQOL-BREF consists of 26 items distributed across four domains—physical health, psychological well-being, social relationships, and environment—and two general questions on overall QoL and health satisfaction.

Responses were scored on a five-point Likert scale ranging from 1 (never) to 5 (always). The mean score for each domain reflects the caregiver's level of satisfaction in that area. Domain scores were transformed to a 0-100 scale, with higher scores indicating better perceived QoL. No specific cutoff points were used to classify QoL as "good" or "poor."^(26,28)^

### Caregiver burden assessment (Zarit Burden Interview)

Perceived caregiver burden was measured using the Zarit Burden Interview (ZBI) also referred to as BI in some studies, which has been translated, culturally adapted, and validated for use in Brazil.^(29)^ This instrument was originally designed to assess burden among caregivers of individuals with functional or behavioral impairments. The ZBI comprises 22 items addressing physical and emotional health, social and personal life, financial situation, and interpersonal relationships.

Each item is rated on a five-point Likert scale ranging from 0 (never) to 4 (always). The total score, obtained by summing all item responses, reflects the overall level of caregiver burden. Based on established thresholds, scores were categorized as follows: little or no burden, 0-21; mild to moderate burden, 21-40; moderate to severe burden, 41-60; and severe burden, 61-88.^(11,26)^

### Statistical analysis

Demographic characteristics, WHOQOL-BREF scores, and caregiver burden data were tabulated and analyzed using *jamovi* software (version 2.5.5; The Jamovi Project, Sydney, Australia) and *BioEstat* (version 5.3; Mamirauá Institute, Belém, Brazil). Descriptive statistics were expressed as frequencies and percentages for categorical variables and as means±standard deviations (SDs) or medians with interquartile ranges (IQRs) for continuous variables, depending on data distribution.

Categorical variables were compared between groups using the Chi-square test or Fisher's exact test, as appropriate. Continuous variables were analyzed using the Mann-Whitney U test for nonparametric data and Student's t-test for normally distributed data. The Wilcoxon test was used to analyze within-group variations in burden scores. Caregiver burden levels were also analyzed according to percentage distribution and evaluated using the G-test for independence. Binary logistic regression analyses were conducted to examine associations between caregiver burden level, categorized as low versus high, and selected variables, including diagnostic group, general dependence, and oral hygiene dependence. The model was intentionally restricted to a limited number of predictors to reduce the risk of overfitting. No adjustment was performed for potential sociodemographic confounders, such as caregiver age, sex, education, or income. Odds ratios (ORs) and 95% confidence intervals (95%CIs) were calculated for each predictor variable. A significance level of 5% (p<0.05) was adopted for all statistical tests.

Item-level analyses of WHOQOL-BREF and ZBI responses were conducted for exploratory purposes. Given the number of comparisons, no formal adjustment for multiple testing was applied; therefore, these results should be interpreted cautiously because of the increased risk of Type I error.

Although sociodemographic variables, including caregiver age, sex, education level, and household income, were collected, they were not included in the regression model because of sample size constraints and the need to limit the number of predictors.

## RESULTS

A total of 124 caregivers or legal guardians participated in the study (Figure 1). The mean age of the children was 9.45 years in the ASD Group and 8.29 years in the NT Group. Sex distribution differed significantly between groups: 80.6% of children in the ASD Group were boys, compared with 45.2% in the NT Group (p<0.001).

**Figure 1:**
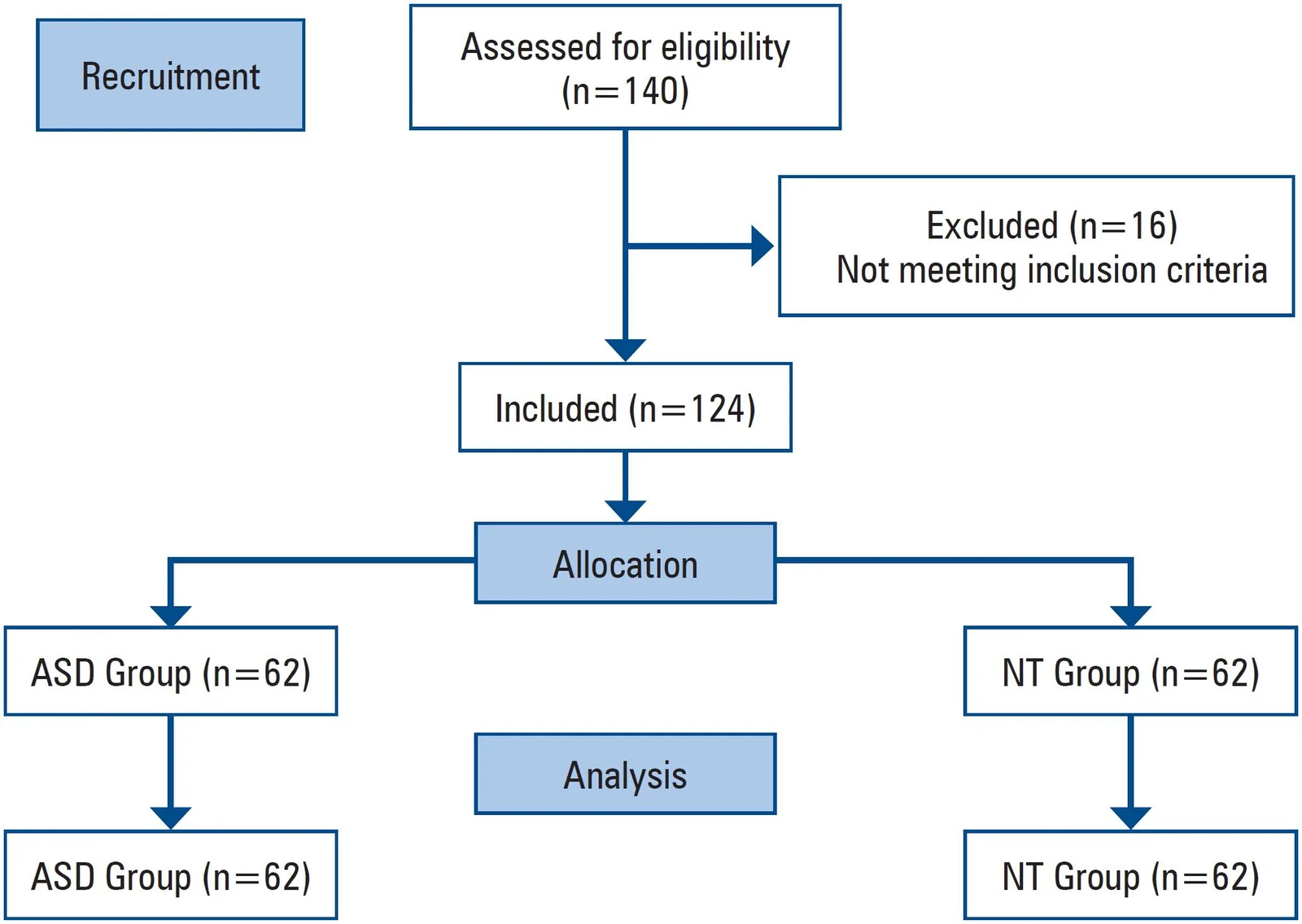
Study design process

Regarding clinical severity, children with ASD were classified by diagnostic level: 50.00% were classified as level 1, indicating a need for mild support; 35.48% were classified as level 2, indicating a need for moderate support; and 14.52% were classified as level 3, indicating a need for substantial or intensive support. These classifications characterize the level of support required by the children with ASD.

Functional dependence differed between groups. In the ASD Group, 69.4% of children depended on caregivers for activities of daily living, whereas 67.7% of children in the NT Group were fully independent (p<0.001). Similarly, 64.4% of children in the ASD Group required caregiver assistance with oral hygiene, whereas 82.3% of NT children performed oral hygiene independently (p<0.001). These findings indicate greater dependence in activities of daily living and oral hygiene among children with ASD than among NT children.

### Caregiver quality of life

Quality of life was assessed using the WHOQOL-BREF questionnaire. Mean scores for each domain—physical health, psychological well-being, social relationships, and environment—and overall QoL were calculated and compared between groups. Overall QoL did not differ significantly between caregivers of children with ASD and caregivers of NT children (p>0.05; Table 1). However, item-level analyses showed differences in selected WHOQOL-BREF items.

**Table 1:** WHOQOL-BREF scores of caregivers or guardians across questionnaire domains

WHOQOL-BREF questionnaire variables, mean (SD)	ASD Group, =62	NT Group, n=62	p value
Domain 1	13.0 (±3.15)	13.6 (±4.23)	0.572
Domain 2	13.0 (±3.08)	13.1 (±4.06)	0.930
Domain 3	12.2 (±3.51)	12.5 (±5.19)	0.738
Domain 4	12.0 (±2.78)	11.9 (±4.64)	0.875
Domain 5	12.2 (±3.46)	11.8 (±4.93)	0.435
Total	12.6 (±2.56)	12.7 (±4.24)	0.944

Domains were assessed using the Mann-Whitney U test, with significance set at p<0.05. ASD: autism spectrum disorder; NT: neurotypical; SD: standard deviation.

Given the number of comparisons performed and the small to moderate effect sizes, the item-level findings should be interpreted cautiously.

As shown in table 2, three items differed significantly between groups, with effect sizes ranging from small to moderate. Q3 showed a small effect (p=0.021; d=0.42), whereas Q4 (p=0.001; d=0.74) and Q26 (p=0.003; d=0.53) showed moderate effects. Thus, although overall QoL scores were similar between groups, selected physical and psychological items differed among caregivers of children with ASD and caregivers of NT children.

**Table 2:** Scores for the 26 WHOQOL-BREF items and corresponding Cohen's d values

Question variables, mean (SD)	ASD Group, n=62	NT Group, n=62	p value	Cohen's d
Q1	3.08 (±0.95)	2.97 (±1.23)	0.578	0.10
Q2	3.03 (±1.07)	2.92 (±1.35)	0.616	0.09
Q3	2.42 (±1.05)	1.98 (±1.05)	0.021	0.42
Q4	2.61 (±1.00)	1.90 (±0.92)	0.001	0.74
Q5	2.98 (±1.12)	2.98 (±1.14)	1.000	0.00
Q6	3.58 (±1.05)	3.50 (±1.46)	0.726	0.06
Q7	3.24 (±1.08)	2.98 (±1.37)	0.243	0.21
Q8	3.37 (±1.18)	3.05 (±1.32)	0.157	0.26
Q9	3.35 (±1.24)	3.27 (±1.38)	0.734	0.06
Q10	3.23 (±1.21)	3.02 (±1.42)	0.377	0.16
Q11	3.21 (±1.28)	3.10 (±1.50)	0.661	0.09
Q12	2.34 (±1.07)	2.35 (±1.20)	0.961	0.01
Q13	2.97 (±1.06)	3.10 (±1.41)	0.562	-0.10
Q14	2.61 (±1.21)	2.55 (±1.24)	0.785	0.05
Q15	3.42 (±1.25)	3.53 (±1.66)	0.677	-0.08
Q16	2.87 (±1.21)	2.87 (±1.43)	1.000	0.00
Q17	3.13 (±1.21)	3.08 (±1.53)	0.840	0.04
Q18	3.19 (±1.23)	3.15 (±1.56)	0.874	0.03
Q19	3.10 (±1.20)	3.15 (±1.39)	0.830	-0.04
Q20	3.08 (±1.06)	3.34 (±1.44)	0.254	-0.20
Q21	2.92 (±1.32)	3.10 (±1.39)	0.461	-0.14
Q22	3.13 (±1.09)	2.97 (±1.41)	0.481	0.13
Q23	3.21 (±1.19)	3.27 (±1.54)	0.808	-0.04
Q24	3.10 (±1.21)	2.82 (±1.45)	0.245	0.22
Q25	3.15 (±1.25)	3.31 (±1.46)	0.513	-0.12
Q26	2.68 (±1.11)	2.10 (±1.08)	0.003	0.53

Items were evaluated using Student's t-test, with significance set at p<0.05. ASD: autism spectrum disorder; NT: neurotypical; Q: question; SD: standard deviation.

### Caregiver burden

Caregiver burden, measured using the ZBI, differed substantially between groups. The mean total burden score was significantly higher among caregivers of children with ASD than among caregivers of NT children (p<0.001; Table 3). When burden levels were categorized as little or none, mild to moderate, moderate to severe, or severe, group differences were also significant (p<0.001), with a higher proportion of caregivers in the ASD Group classified as having moderate to severe or severe burden.

**Table 3:** Caregiver burden scores among caregivers or guardians

Caregiver burden variables	ASD Group, n=62	NT Group, n=62	p value
Total burden score, mean (SD)	34.3 (±15.0)	16.6 (±10.7)	<0.001
Caregiver burden categories - n (%)			
Little or none	11 (17.7)	47 (75.8)	
Mild to moderate	31 (50)	13 (21)	<0.001
Moderate to severe	16 (25.8)	2 (3.2)	
Severe	4 (6.5)	-	

The total score was assessed using the Wilcoxon test, and burden categories were assessed using the G-test for independence, with significance set at p<0.05. ASD: autism spectrum disorder; NT: neurotypical; SD: standard deviation.

Item-level analyses of caregiver burden showed significant differences for most items. Among the 22 items assessed, only Q4 and Q5 did not differ significantly between groups (p>0.05). All other items differed significantly between groups (p<0.05; Table 4).

**Table 4:** Item-level scores on the caregiver burden questionnaire

Question variables, median (IQR)	ASD Group, n=62	NT Group, n=62	p value
Q1	2.00 (2.00-3.00)	1.00 (0.00-2.00)	<0.001
Q2	2.00 (2.00-3.00)	1.00 (0.00-2.00)	<0.001
Q3	2.00 (1.00-3.00)	1.00 (0.00-2.00)	0.004
Q4	2.00 (2.00-3.00)	1.00 (0.00-2.00)	0.233
Q5	0.00 (0.00-1.00)	0.00 (0.00-0.00)	0.107
Q6	0.00 (0.00-2.00)	0.00 (0.00-0.00)	0.002
Q7	2.00 (2.00-4.00)	1.00 (0.00-2.00)	<0.001
Q8	3.00 (2.00-4.00)	2.00 (1.00-3.00)	<0.001
Q9	0.00 (0.00-2.00)	0.00 (0.00-0.00)	0.004
Q10	1.00 (0.00-2.00)	0.00 (0.00-0.00)	<0.001
Q11	1.00 (0.00-2.00)	0.00 (0.00-1.00)	0.005
Q12	1.00 (0.00-2.00)	0.00 (0.00-0.00)	<0.001
Q13	0.00 (0.00-2.00)	0.00 (0.00-0.00)	0.009
Q14	2.00 (1.00-3.00)	1.00 (0.00-2.00)	0.002
Q15	3.00 (2.00-4.00)	1.00 (0.00-2.00)	<0.001
Q16	1.00 (0.00-2.00)	0.00 (0.00-0.00)	<0.001
Q17	0.00 (0.00-2.00)	0.00 (0.00-0.00)	0.008
Q18	0.00 (0.00-1.00)	0.00 (0.00-0.00)	0.038
Q19	2.00 (0.00-2.00)	0.00 (0.00-1.00)	<0.001
Q20	2.00 (2.00-4.00)	2.00 (0.00-3.00)	0.002
Q21	2.00 (1.00-3.00)	1.00 (0.00-2.75)	0.009
Q22	2.00 (1.00-3.00)	1.00 (0.00-1.75)	<0.001

Questions were evaluated using the Mann-Whitney U test (p<0.05). ASD: autism spectrum disorder; NT: neurotypical; Q: question; IQR: interquartile range.

Binary logistic regression analysis showed a significant association between group and caregiver burden category (ASD *versus* NT: OR=0.11; 95%CI: 0.02-0.55; p=0.008). Other variables, including general functional dependence and oral hygiene dependence, were not significantly associated with caregiver burden category (p>0.05; Table 5). This association should be interpreted cautiously because adjustment for potential confounders was limited.

**Table 5:** Binomial logistic regression analysis of caregiver burden category: little or mild burden versus moderate or severe burden

		Caregiver burden category, OR (95%CI)	p value
General dependence			
	Independent	Ref	
	Dependent	0.86 (0.22-3.34)	0.830
Oral hygiene dependence			
	Independent	Ref	
	Dependent	1.88 (0.48-7.33)	0.361
Groups			
	NT	Ref	
	ASD	0.11 (0.02-0.55)	0.008

Questions were evaluated using the Mann-Whitney U test (p<0.05). ASD: autism spectrum disorder, NT: neurotypical, Q: question, IQR: interquartile range.

Collectively, these results show higher caregiver burden among caregivers of children with ASD, despite similar overall perceived QoL between caregivers of children with ASD and caregivers of NT children. These findings support the need for targeted programs addressing caregiver burden and caregiver well-being.

## DISCUSSION

This study analyzed QoL and caregiver burden among caregivers of children with ASD and those of NT children. Our findings are consistent with several patterns reported in the literature, particularly the higher proportion of boys in the ASD Group.^(30)^ This distribution is consistent with epidemiological data showing a male predominance in ASD diagnoses. ASD is associated with behavioral and cognitive impairments that can affect development, learning, and independence and may require continuous caregiver support.^(31)^ Consistent with previous reports, children with ASD in this study showed higher levels of dependence in activities of daily living and oral hygiene.^(11,26)^ This elevated dependence may increase caregiving demands on parents and guardians, requiring additional time, effort, and emotional resources.

A key contribution of this study is its evaluation of caregiver burden within a specialized dental care setting, an aspect that remains underexplored. Previous studies^(19-25)^ have reported increased burden among caregivers of children with ASD, but most have focused on general health or psychosocial contexts. In the dental care context, additional demands may arise from oral hygiene assistance and the management of behavioral challenges during clinical procedures. Because both groups were recruited from the same institution, this design may have reduced variability related to healthcare access and allowed a more focused comparison of ASD-related caregiving demands.

The predominance of mothers as primary caregivers was also evident in our sample, reflecting a trend reported across different cultural contexts.^(10,32-35)^ Despite societal shifts in gender roles, women often continue to assume most caregiving responsibilities, whereas fathers more frequently focus on financial provision.^(33,36)^ This pattern supports the need for interventions that address the challenges faced by mothers while promoting equitable support for both parents. Primary caregivers also play an important role in supporting social inclusion among children with disabilities and promoting their integration into the community. However, extensive caregiving responsibilities, particularly when undertaken with limited institutional or social support, can adversely affect caregivers’ personal and family life and reduce overall QoL.^(16,37)^

In this study, overall QoL scores^(27,38)^ did not differ significantly between caregivers of children with ASD and caregivers of NT children, suggesting that broad perceptions of QoL may be similar between groups. Although item-level analyses identified differences in specific physical and psychological aspects, these findings should be interpreted cautiously. These analyses involved multiple comparisons without formal adjustment, increasing the risk of Type I error, and the observed effect sizes were small to moderate. Therefore, the absence of differences in global QoL should be emphasized because it provides a more robust measure of overall well-being than individual item responses. This distinction is important when interpreting group differences in QoL in cross-sectional studies.

Several factors may explain the absence of significant differences in global QoL between caregivers of children with ASD and caregivers of NT children. First, caregivers of children with ASD may develop adaptive coping strategies over time, including psychological resilience, reorganization of daily routines, and reliance on informal or formal support networks, which may mitigate the effect of caregiving demands on overall life perception. Second, participants were recruited from a specialized multiprofessional care setting, which may have provided structured support, guidance, and access to healthcare resources that buffered some negative effects of caregiving. Third, global QoL measures such as the WHOQOL-BREF may lack sensitivity to detect subtle, domain-specific differences, as suggested by the item-level differences observed. Finally, caregivers of NT children may also experience stress related to parenting, daily responsibilities, and socioeconomic factors, which may reduce between-group differences when broad QoL measures are used. Together, these factors may explain why overall QoL scores were similar despite higher caregiver burden in the ASD Group.

Consistent with previous studies, caregiver burden was significantly higher among caregivers of children with ASD than among caregivers of NT children. Most caregivers in the ASD Group reported mild to moderate burden, although individual experiences varied.^(11,26,33,39-44)^ Elevated burden may have multiple implications, including depressive and anxiety symptoms, emotional exhaustion, social isolation, reduced quality of care, absenteeism, and decreased productivity. Regression analysis showed an association between diagnostic group and caregiver burden category, suggesting that ASD-related caregiving demands may be important contributors to burden. This finding is relevant to dental care because caregiver burden may reflect not only the practical demands of oral hygiene assistance but also broader behavioral, emotional, and organizational challenges associated with ASD that may become more prominent during oral health management.

Caregiver burden is closely linked to emotional distress and social limitations, supporting the need for interventions that address both caregiver well-being and child-related care needs. Behavioral challenges in children with ASD further increase this burden, supporting integrated approaches that address family well-being alongside child-focused interventions.^(45-47)^ The specialized, multidisciplinary care provided in the study setting may also have supported oral health maintenance and alleviated some caregiving challenges. Nonetheless, regional disparities in access to healthcare and social services can affect caregiver QoL and exacerbate burden, highlighting the need for comprehensive support policies. Initiatives such as the Integrated State Plan for People with ASD (PEIPTEA) in São Paulo exemplify intersectoral strategies aimed at enhancing inclusion, protecting rights, and improving QoL for individuals with ASD and their families.

From a clinical perspective, these findings support a family-centered approach to dental care for individuals with ASD. Strategies that reduce procedural stress, improve patient cooperation, and support caregivers—such as behavioral guidance techniques, caregiver education, and individualized treatment planning—may improve oral health outcomes and reduce caregiver burden. Thus, dental professionals may play an important role not only in managing oral conditions but also in supporting the broader well-being of families affected by ASD.

Caregiver burden is influenced by psychosocial and socioeconomic factors. In this study, perceived social support, caregiver mental health history, and coping strategies were not assessed; moreover, although sociodemographic characteristics were collected, they were not incorporated into the regression model. These factors may have contributed to the observed outcomes. Their absence limits the ability to account for potential confounding and restricts the interpretation of the regression findings.

This study has several limitations that should be considered. First, data were self-reported and may be subject to recall bias or social desirability effects. Second, variables such as duration of caregiving, caregiver physical and mental health, social support, and socioeconomic status were not controlled, and the study was conducted in a specific geographic region, limiting generalizability. Third, participants were recruited from a specialized multiprofessional dental care center, which may limit generalizability because these caregivers likely had greater access to structured healthcare services and professional support than the broader population of caregivers of children with ASD. This context may have influenced both perceived burden and coping strategies. In addition, although caregivers were recruited consecutively, the clinical setting reflects a convenience-based population of individuals actively seeking care. Furthermore, excluding caregivers with diagnosed psychiatric disorders, although intended to reduce confounding in self-reported outcomes, may have led to underestimation of caregiver burden because individuals with higher levels of psychological distress were not represented in the sample. Therefore, caution is warranted when generalizing these findings to populations with limited access to multidisciplinary care or higher levels of psychological vulnerability. Despite these limitations, the findings support targeted, context-specific support strategies, particularly within dental care settings, to reduce caregiver stress and improve well-being in families affected by ASD.

Additional methodological considerations should also be acknowledged. The sample size calculation was based on differences in QoL scores measured using the WHOQOL-BREF, and no specific power analysis was conducted for caregiver burden or logistic regression analyses. Although the study detected substantial between-group differences in caregiver burden, the regression analyses included only a limited number of predictors and did not include key sociodemographic or psychosocial variables, such as caregiver age, sex, education, income, or social support. The item-level analyses also involved multiple comparisons without formal correction, increasing the risk of Type I error. Therefore, these item-level and regression findings should be interpreted cautiously. Finally, the relatively modest sample size may restrict the stability and generalizability of multivariable estimates; therefore, future studies with larger samples are warranted to confirm these associations. Future studies should incorporate comprehensive multivariable models that include psychosocial, behavioral, and socioeconomic factors to better characterize the determinants of caregiver burden and identify potential targets for intervention.

## CONCLUSION

Caregivers of children with autism spectrum disorder and caregivers of neurotypical children generally reported moderate quality of life. However, caregivers of children with autism spectrum disorder experienced significantly higher caregiver burden, supporting the need for targeted strategies to address caregiving-related stress and support caregiver well-being.

## Data Availability

After publication, data will be available from the authors upon request—this condition is justified in the manuscript.
